# Target protein-oriented isolation of Hes1 dimer inhibitors using protein based methods

**DOI:** 10.1038/s41598-020-58451-3

**Published:** 2020-01-28

**Authors:** Midori A. Arai, Kaori Morita, Haruka Kawano, Yuna Makita, Manami Hashimoto, Akiko Suganami, Yutaka Tamura, Samir K. Sadhu, Firoj Ahmed, Masami Ishibashi

**Affiliations:** 1 Graduate School of Pharmaceutical Sciences, Chiba University, 1-8-1 Inohana, Chuo-ku, Chiba 260-8675 Japan; 2 Graduate School of Medicine, Chiba University, 1-8-1 Inohana, Chuo-ku, Chiba 260-8670 Japan; 3 Pharmacy Discipline, Khulna University, Khulna, 9208 Bangladesh; 4 Department of Pharmaceutical Chemistry, University of Dhaka, Dhaka, 1000 Bangladesh

**Keywords:** Neural stem cells, Natural products

## Abstract

Natural products isolation using protein based methods is an attractive for obtaining bioactive compounds. To discover neural stem cell (NSC) differentiation activators, we isolated eight inhibitors of Hes1 dimer formation from *Psidium guajava* using the Hes1-Hes1 interaction fluorescent plate assay and one inhibitor from *Terminalia chebula* using the Hes1-immobilized beads method. Of the isolated compounds, gallic acid (**8**) and 4-*O*-(4”-*O*-galloyl-α-L-rhamnopyranosyl)ellagic acid (**11**) showed potent Hes1 dimer formation inhibitory activity, with IC_50_ values of 10.3 and 2.53 μM, respectively. Compound **11** accelerated the differentiation activity of C17.2 NSC cells dose dependently, increasing the number of neurons with a 125% increase (5 μM) compared to the control.

## Introduction

Neurodegenerative diseases such as dementia and spinal cord injury severely compromise the quality of life. The discovery of neural stem cells (NSCs) in human adult brain^1,2^ led to the expectation of novel treatments involving NSC transplantation or accelerated differentiation of internal NSCs in the brain to overcome neurodegenerative diseases. However, the development of these approaches and small molecule NSC activators has been slow, possibly due to the complex mechanism underlying the differentiation of NSCs to neural cells.

Basic helix-loop-helix (bHLH) factors play crucial roles in the differentiation of neural cells such as neurons, astrocytes and oligodendrocytes^3^. For example, proneural bHLH factors, such as Ascl1 (formerly Mash1), Neurog1 and Neurog2 regulate the generation of neurons. Olig1 and Olig2 are bHLH factors important for the maturation of oligodendrocytes. Hairy and enhancer of split (hes) bHLH factors promote the self-renewal of NSCs and the generation of astrocytes, as well as inhibiting the differentiation of NSCs by suppressing the expression of proneural genes. Recently, Kageyama *et al*. reported that oscillation in the concentration of bHLH factors directly controls the fate of NSCs^4,5^. Hes1 and Ascl1 proteins oscillate with 2- to 3-hour periods, whereas Olig2 oscillates with a 5- to 8-hour period. During cell fate choice, one bHLH factor is expressed in a sustained manner while the others are repressed. In neurons, Ascl1 is expressed sustainably while Hes1 and Olig2 expression is inhibited.

**Figure 1:**
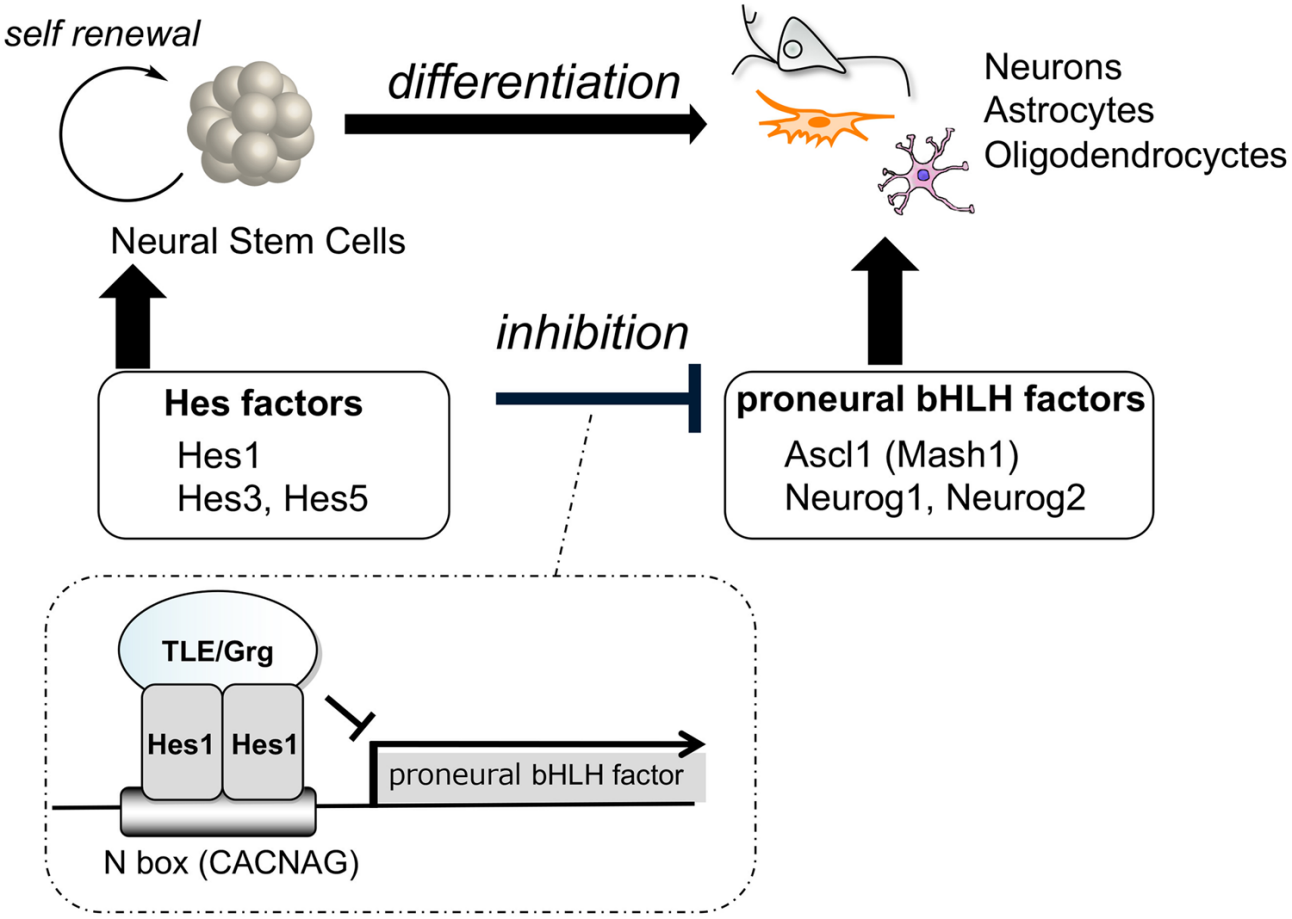
Neural stem cell differentiation and fate control by bHLH factors.

Hes1 dimerizes and binds to the promoter region of Ascl1 and other proneural genes (Fig. 1), making the inhibition of Hes1 an attractive approach to enhance neuronal differentiation. If the interested target protein was decided, it would be faster and effective to isolate the bioactive natural products using a “protein-based assay system”^6^. However, the number of reports of isolation using protein-based assay system was still limited (affinity chromatography^7–9^, protein-immobilized beads^10–18^, ultrafiltration^19–21^, and protein-protein interactions^22,23^). In our attempts to find Hes1 inhibitors effectively, we constructed a new Hes1-Hes1 interaction fluorescent plate assay which gave the first Hes1 dimer inhibitors^24^. Also, we have developed “target protein oriented natural products isolation (TPO-NAPI) methods” using protein-immobilized beads^11,15,17,18^. Lindbladione^15^, agalloside^15^, isomicromonolactam^17^ and inohanamine^17^ were isolated by using Hes1 protein beads and accelerated the differentiation of NSCs to neurons. Here, we report the isolation of eight Hes1 dimer inhibitors from *Psidium guajava* using the Hes1-Hes1 interaction fluorescent plate assay, which is the first example of natural products isolation using fluorescent plate assay of protein-protein interactions (PPIs). Moreover, the isolation using the TPO-NAPI method with Hes1 beads gave a Hes1 dimer inhibitor from *Terminalia chebula*.

## Results and Discussion

**Figure 2:**
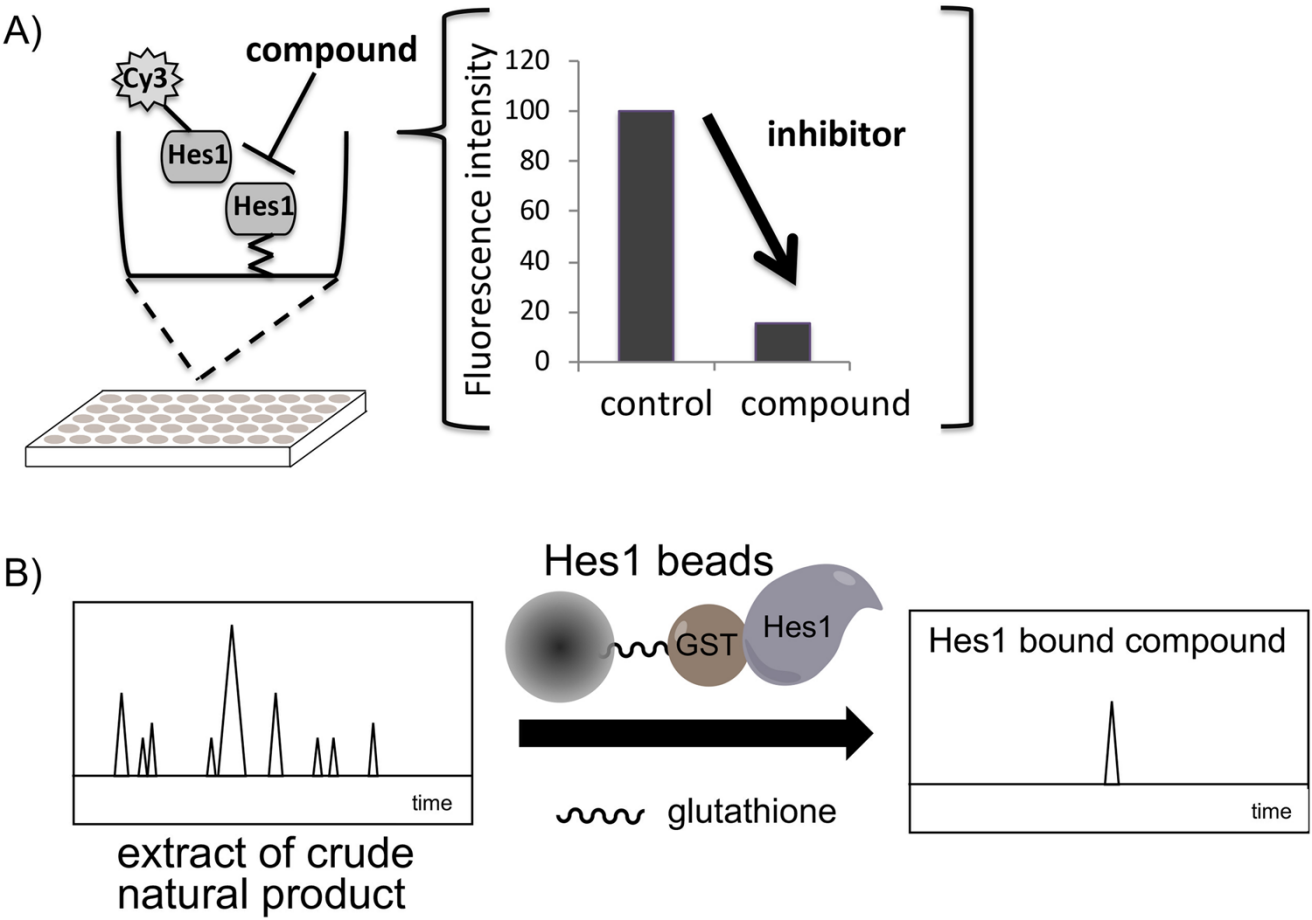
Target protein-oriented isolation methods. (**A**) Hes1-Hes1 interaction fluorescent plate assay, (**B**) Hes1 immobilized beads method.

**Figure 3:**
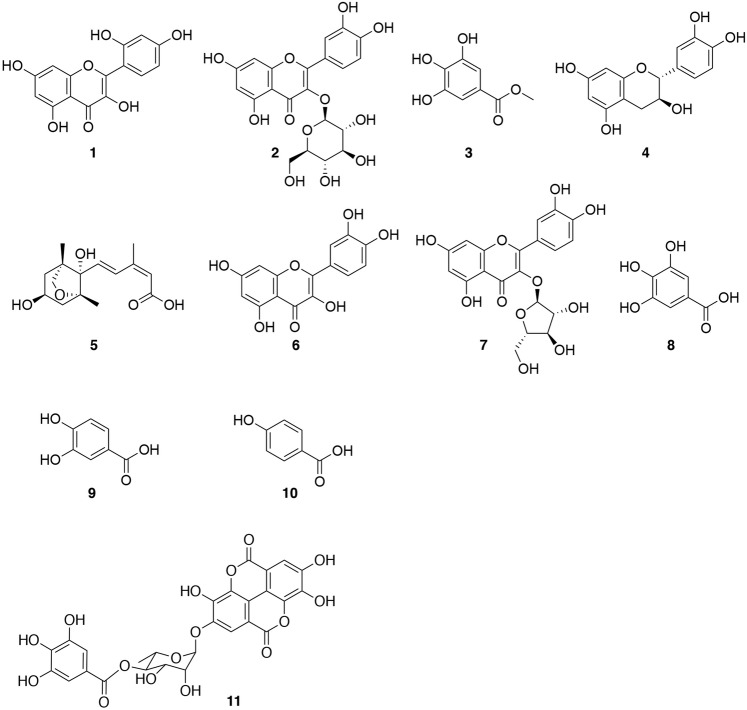
Structures of the isolated compounds.

**Figure 4:**
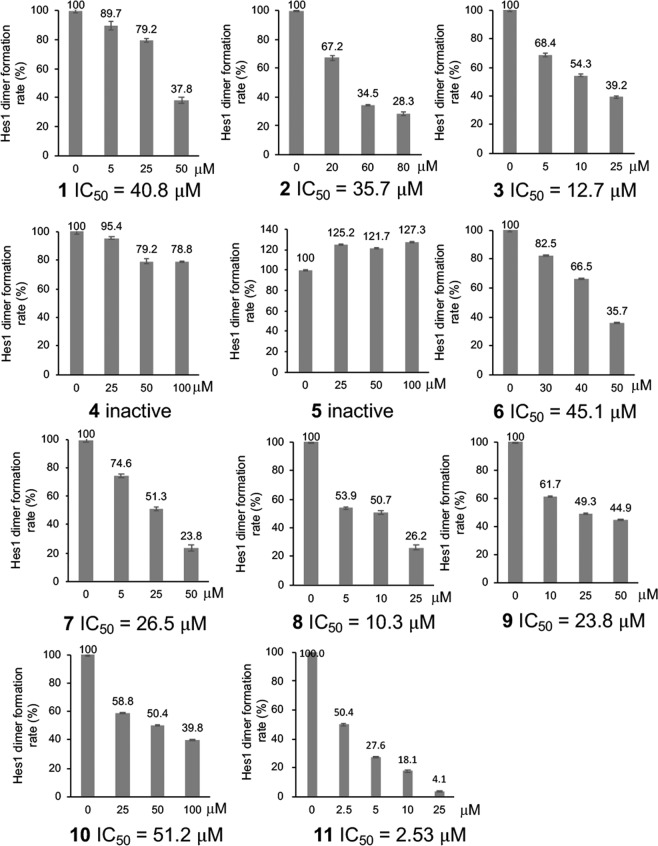
Hes1 dimer formation inhibitory activities of the isolated compounds.

It is essential to inhibit undesired PPIs in drug development, and bioactivity-guided isolation using the inhibitory activity of PPIs is an attractive method to obtain effective inhibitors. However, currently there are only a few examples of such approaches using PPI assay systems^22,23^. We previously developed a Hes1-Hes1 interaction fluorescent plate assay and reported the screening results of our natural products compound library (Fig. 2A)^24^. Glutathione-*S*-transferase (GST) fused rat Hes1(3–281) protein was expressed in *Escherichia coli* and purified rat Hes1 protein was immobilized on the bottom of 96 well plates. We prevented GST-GST interactions, which would result in false positives, by preparing GST-free Hes1 protein by GST cleavage with Turbo3C protease. After labeling Hes1 with Cy3, this florescent Hes1 protein was added to the wells of the above plate and incubated for 24 h at 4 °C. Hes1-Hes1 interaction was successfully detected as Cy3 fluorescence intensity. Using this assay system, we screened our 118 plant extract library and identified the MeOH extract of *Psidium guajava* leaves to contain naturally occurring compounds that inhibit Hes1 dimer formation. The MeOH extract (29.9 g) was fractionated using Diaion HP-20 with a MeOH-acetone solvent system to afford fractions 1A to 1C. Active fraction 1A (27.2 g) was suspended in 10% aq. MeOH and partitioned with hexane, EtOAc and BuOH to obtain hexane (1.1 g), EtOAc (5.7 g), BuOH (4.7 g) and aqueous (18.9 g) soluble fractions. Part of the active BuOH soluble fraction was subjected to ODS column chromatography and reverse-phase HPLC. Activity-guided separation yielded ten compounds (**1**–**10**; Fig. 3). The isolated compounds were identified as morin (**1**)^25^, isoquercitrin (**2**)^26^, methyl gallate (**3**)^27^, (+)-catechin (**4**)^28,29^, dihydrophaseic acid (**5**)^30^, quercetin (**6**)^26,31^, avicularin (**7**)^32,33^, gallic acid (**8**)^34^, protocatechuic acid (**9**)^35^ and 4-hydroxybenzoic acid (**10**)^36^ based on comparisons of their spectral data with spectra in the literature. The Hes1-Hes1 interaction inhibitory activities of the isolated compounds were examined (Fig. 4) and **3**, **7**, **8** and **9** produced moderate inhibition (IC_50_ 12.7, 26.5, 10.3 and 23.8 μM). The most potent inhibitor was gallic acid (**8**). Commercially available gallic acid also exhibited comparable inhibition (IC_50_ 8.9 μM). Inhibition by the gallic acid derivatives **3**, **8**, **9** and **10** showed that the number of phenolic hydroxyl groups affects inhibitory activity, with activity decreasing as the number of phenolic hydroxyl groups decrease.

**Figure 5:**
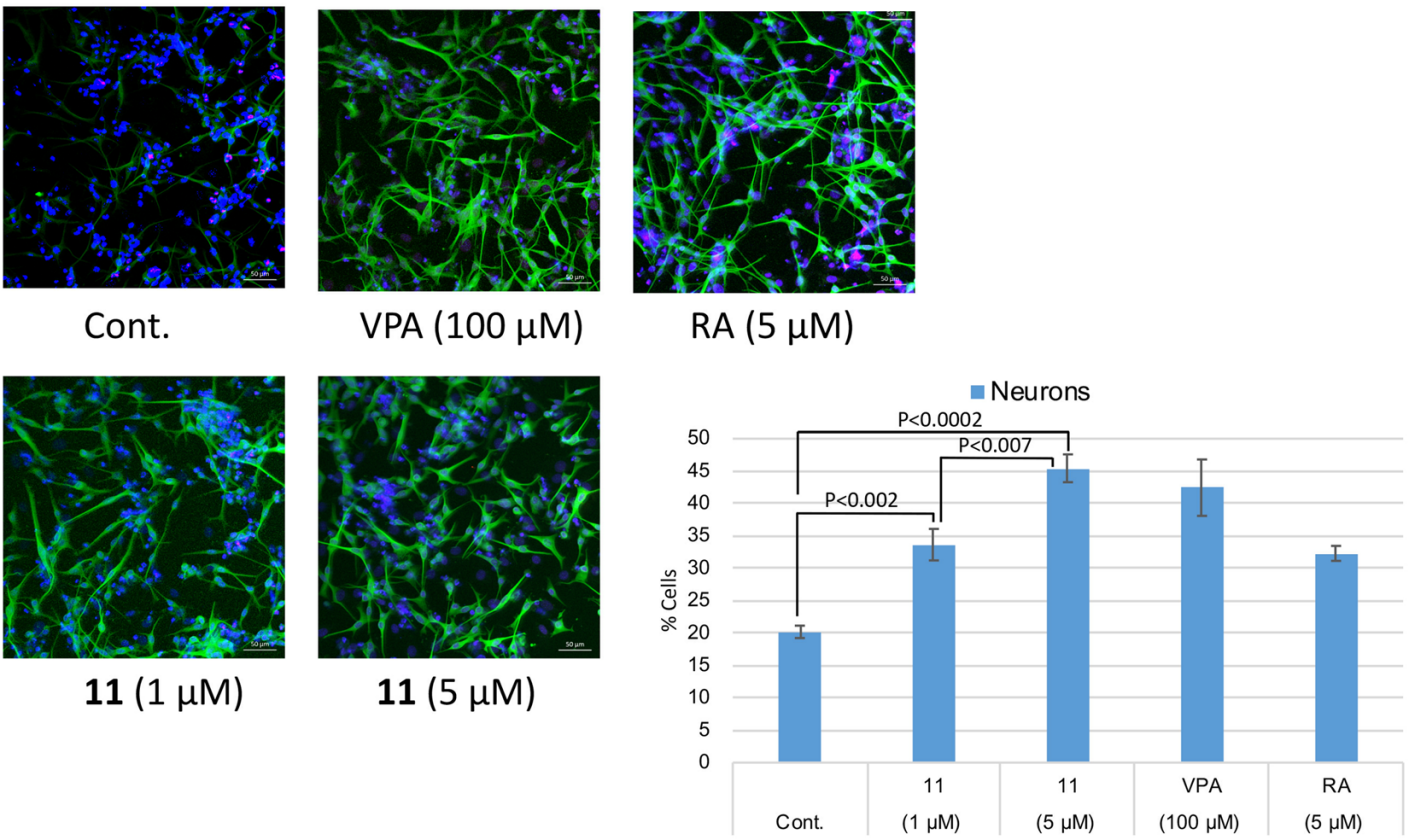
Effect of 11 on NSC differentiation into neurons. Green: neurons, blue: nuclei, red: astrocytes. The number of neurons increased following the addition of compound 11 dose dependently. *P* values were analyzed by Student’s *t* test. *P* < 0.05 is considered statistically significant.

We recently developed another protein-based screening method, the target protein oriented natural products isolation method (TPO-NAPI) using protein beads (Fig. 2B). Agalloside, inohanamine, α-mangostine, BE-14106, isomicromonolactam, staurosporin and linarin were isolated as Hes1 binding compounds using the TPO-NAPI method^15,17^. Rat Hes1 (1–95) containing basic and helix-loop-helix domains was immobilized because the helix-loop-helix domain is known to be important for Hes1-Hes1 interaction; therefore, utilizing this domain in the beads method would likely be effective for screening Hes1 dimer inhibitors. GST-Hes1 immobilized beads were prepared by mixing freshly prepared GST-Hes1 protein with glutathione Sepharose 4B beads. GST-only beads were prepared as a control. After incubating the beads with plant MeOH extracts at 4 °C for 2 h, bound compounds were eluted by adding EtOH and heating at 100 °C for 3 min, then the eluted compounds were analyzed by HPLC. Of the 105 plant MeOH extracts screened using this method, the Bangladesh plant *Terminalia chebula* was found to contain a Hes1 binding compound. The MeOH extract (64.6 g) of *Terminalia chebula* bark was partitioned with hexane, EtOAc and BuOH to obtain hexane (1.5 g), EtOAc (3.6 g), BuOH (42.6 g), and aqueous (20.5 g) soluble fractions. The EtOAc fraction contained the target peak and was subjected to silica gel column chromatography to give eight fractions (1A-H). Fraction 1D contained the target peak and was separated by ODS column chromatography and reverse-phase HPLC to give compound **11** (0.4 mg). Compound **11** was identified as 4-*O*-(4″-*O*-galloyl-α-L-rhamnopyranosyl)ellagic acid by comparison of its NMR data with reported spectral data^37^. Next, we evaluated the Hes1 dimer inhibitory activity of compound **11** (Fig. 4) and found that it exhibited the most potent inhibition of Hes1 dimer formation of the compounds tested, with an IC_50_ value of 2.53 μM. This is the strongest Hes1 dimer inhibitor reported to date. The ability of **11** to accelerate the differentiation of C17.2 mouse neural stem cells was evaluated (Fig. 5). C17.2 cells (2 × 10^5^ cells/mL) were seeded in a poly-L-lysine-coated 24 well plates and incubated for 24 h, then the cells were treated with DMSO (control), 100 μM valproic acid (VPA), 5 μM retinoic acid (RA) (positive controls) or **11** (1 or 5 μM) for 6 days. A confocal microscope was used to obtain images of differentiated neural cells after immunostaining class III β-tubulin (Tuj1) in neurons, and glial fibrillary acidic protein (GFAP) in astrocytes and nuclei (TO-PRO-3). The number of neurons increased following the addition of VPA, RA or compound **11**, suggesting that these compounds enhanced the number of C17.2 neurons. In contrast, the control dish containing cells treated with DMSO contained small number of neurons. The differentiated neurons with **11** were 33.6% (1 µM) and 45.4% (5 µM), which are 67% and 125% increase compared to those of control. These results suggested that compound **11** accelerates the differentiation of NSCs to neurons by inhibiting Hes1 dimer formation.

**Figure 6:**
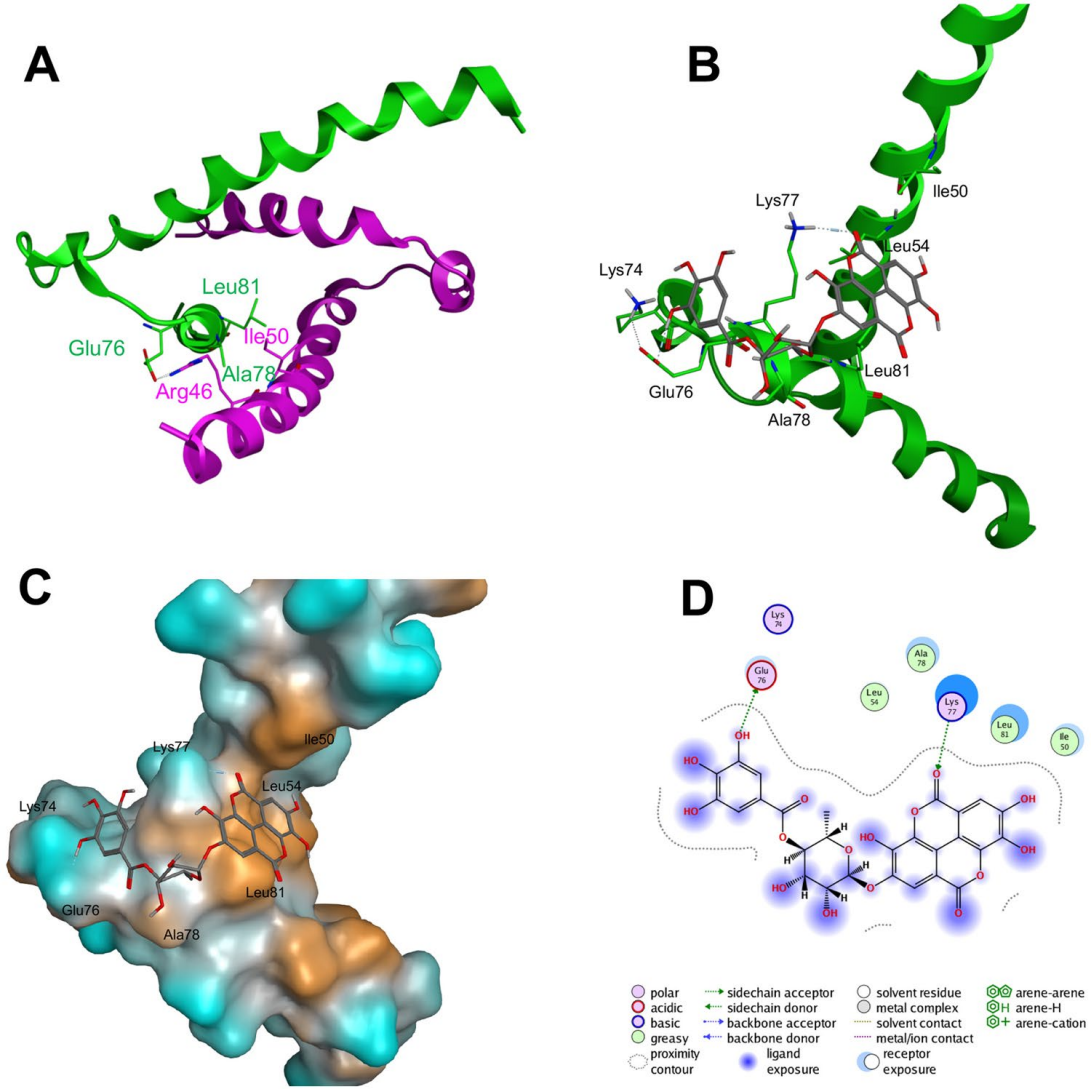
Docking study of compound 11 to the Hes1 HLH domain. (**A**) NMR structure of the HLH domains of Hes1 dimers (PDB code: 2MH3). Green and pink show individual Hes1 HLH domains. (**B**–**D**) Docking study results of 11 with the HLH domain.

We also predicted the interaction between the HLH domain of Hes1 protein and compound **11** (4-*O*-(4″-*O*-galloyl-α-L-rhamnopyranosyl)ellagic acid) by performing *in silico* docking analysis of compound **11** with the HLH domain of Hes1. As shown in Fig. 6A,B, the galloyl site of compound **11** might interact with the loop region of Hes1, aiding the formation of Hes1(Arg46 of helix region)-Hes1(Glu76 of loop region) and preventing mutual recognition by Hes1 molecules. On the other hand, the ellagic acid site of compound **11** might bind with the helix region of Hes1, which consists of Ile50, Leu54 and Leu81, preventing hydrophobic core formation in the Hes1 dimer. Orange shows the hydrophobic region in Hes1 (Fig. 6C). Moreover, hydrogen bond formation between the ellagic acid site of compound **11** with Lys77 might obstruct Hes1(loop region)-Hes1(loop region) formation. Blue shows the hydrophilic region. As shown in Fig. 6C, the interaction of galloyl moiety with the hydrophilic region seems to be important. Therefore, the decrease of inhibition with decrease of number of phenolic hydroxyl groups in galloyl group would be reasonable. In addition, the α-L-rhamnopyranosyl unit appears to be an efficient linker, enabling tight interaction between compound **11** with the Hes1 monomer via its galloyl and ellagic acid sites.

## Conclusion

In conclusion, several Hes1 binding natural products were isolated using two target protein-oriented isolation methods: a Hes1-Hes1 interaction fluorescent plate assay and a Hes1-immobilized beads method. Ten compounds were isolated from *Psidium guajava*, of which eight showed Hes1 dimer inhibitory activity. 4-*O*-(4″-*O*-Galloyl-α-L-rhamnopyranosyl)ellagic acid (**11**) isolated using the Hes1 beads method showed potent Hes1 dimer formation inhibitory activity, with an IC_50_ of 2.53 μM. Compound **11** accelerated the differentiation of C17.2 NSC cells into neurons dose dependently, increasing the number of neurons with a 125% increase (5 μM) compared to the control. There are few reports of Hes1 dimer inhibitors and thus these results will be useful for evaluating Hes1-related mechanisms in cells. We believe that protein-based isolation is an effective approach for identifying bioactive compounds.

## Methods

### General experimental procedures

NMR spectra were recorded on JEOL ECZ600 spectrometers in a deuterated solvent whose chemical shift was used as an internal standard. Column chromatography was performed using Silica Gel 60N (Kanto Chemical Co., Tokyo, Japan), Chromatorex ODS (Fuji Silysia Chemical Ltd., Kasugai, Japan), Diaion HP-20 (Mitsubishi Chemical Co., Tokyo, Japan) and Sephadex LH-20 (GE Healthcare Japan, Tokyo, Japan). Preparative HPLC was performed using YMC-Pack SIL-06 (YMC Co. Ltd., Kyoto, Japan), COSMOSIL 5C_18_-AR-II, COSMOSIL Cholester, COSMOSIL πNAP (Nacalai Tesque, Inc. Kyoto, Japan) and CAPCELL PAK C_18_ MGII (OSAKA SODA Ltd., Osaka, Japan).

### Plant materials

*Psidium guajava* (Myrtaceae) and *Terminalia chebula* (Combretaceae) were collected in Bangladesh in 2013 and were identified by one of the authors (S. K. Sadhu). Voucher specimens (KKB348 and KKB364) were deposited at the Department of Natural Products Chemistry, Graduate School of Pharmaceutical Sciences, Chiba University.

### Plate assay for Hes1 dimer inhibitors

Hes1-bound microplate wells^24^ (Nunc Immobilizer^TM^ Amino Plate, Thermo) were incubated with 50 μL of Cy3-labeled-Hes1 in NET-N buffer (NET buffer (20 mm Tris-HCl, pH 7.5, 200 mm NaCl, 1 mm EDTA) containing 0.05% Nonidet^®^ P-40, ca. 7 mg/L, dye/protein = 0.4) for 24 h at 4 °C. After removing the protein solution, the wells were washed twice with 200 μL of PBST, then each compound solution (in NET-N buffer, 50 μL) was added and the plates were incubated for 1 h at RT in the dark. The wells were washed twice with 200 μL of PBST, then dried under reduced pressure for 1 h in the dark. The Cy3 dye was excited at 544 nm and emission was monitored at 590 nm using a microplate reader (Fluoroskan Ascent, Thermo Labsystems, Vantaa, Finland). The assays were typically carried out in three individual wells, and the mean value and SD were calculated. The equilibrium between dimer formation between Cy3-Hes1/Cy3-Hes1 (in solution) and Cy3-Hes1/immobilized-Hes1 allowed the detection of Cy3-Hes1/immobilized-Hes1.

### Typical screening procedure for the Hes1 beads method

To prepare GST-Hes1 beads, GST-ratHes1_1-95_ (ca. 3.6 nmol) in PBS was added to pre-washed glutathione Sepharose 4B beads (bed volume 100 μL, GE Healthcare) and mixed at 4 °C for 1 h. The GST-Hes1 beads were washed five times with NET buffer, then suspended in 250 μL NET buffer. A MeOH or EtOAc plant extract (125 μg in EtOH, 25 μL) was added to the above GST-Hes1 freshly prepared beads (bed volume 100 μL) and gently mixed for 2 h at 4 °C. The beads were then washed by tapping the container with fingers three times with NET-N buffer. EtOH (70%, 150 μL) was added to the washed beads and the suspension was heated at 100 °C for 3 min in a heating block. The beads were collected by centrifugation (2000 rpm, 4 °C, 1 min) and the supernatant was centrifuged at 15000 rpm for 15 min. One-third of the supernatant was analyzed by HPLC. Control GST-beads were prepared in the same manner as GST-Hes1 beads by adding GST (ca. 3.8 nmol) in PBS to pre-washed glutathione Sepharose 4B beads (bed volume 100 μL, GE Healthcare). Lindobladione was used as a positive control in each experiment. Obvious differences in peak intensity for the results obtained using GST-Hes1-beads and GST-beads (control) were considered “hit” extracts containing a Hes1 binding natural product.

### Extraction and isolation

The MeOH extract (29.9 g) of *Psidium guajava* (leaves; 139 g) was subjected to Diaion HP-20 (φ 70 × 210 mm) chromatography with a MeOH-acetone solvent system (1:0–0:1) to afford fractions 1A to 1 C. Active fraction 1A (27.2 g) was suspended in 10% aq. MeOH (150 mL) and partitioned with hexane, AcOEt and BuOH (400 mL × 3) to obtain hexane (1.1 g), AcOEt (5.7 g), BuOH (4.7 g) and aqueous (18.9 g) soluble fractions. An aliquot of the active BuOH soluble fraction was subjected to ODS column chromatography (φ 40 × 210 mm; MeOH-H_2_O = 3:7–1:0) to afford fractions 3A to 3E. Fraction 3B (134.6 mg) was subjected to ODS HPLC (COSMOSIL 5C_18_-AR-II; 40% MeOH + 0.1% HCOOH, flow rate 3.0 mL/min) to give compound **1** (4.6 mg). An aliquot of fraction 3D (124.5 mg) was subjected to ODS HPLC (COSMOSIL 5C_18_-AR-II; 40% MeOH + 0.1% HCOOH, flow rate 5.0 mL/min) to give compound **2** (7.3 mg). An aliquot of the active AcOEt soluble fraction was subjected to ODS column chromatography (φ 40 × 210 mm; MeOH-H_2_O = 3:7–1:0) to afford fractions 2A to 2I. Fraction 2B (236.5 mg) was subjected to ODS column chromatography (φ 20 × 460 mm; MeOH-H_2_O = 4:6–1:0) to give compound **3** (10.9 mg) and to afford fractions 4A to 4E. Fraction 4 C (32.9 mg) was subjected to silica gel column chromatography (φ 20 × 280 mm; CHCl_3_-MeOH = 30:1-0:1) to give compound **4** (10.3 mg). Fraction 4A (10.0 mg) was subjected to HPLC (YMC-Pack SIL-06, CHCl_3_-MeOH = 6:1, flow rate 3.0 mL/min) to give compound **5** (0.3 mg). Fraction 2 H (83.3 mg) was subjected to silica gel column chromatography (φ 15 × 310 mm; AcOEt-MeOH = 150:1–0:1) to give compound **6** (21.6 mg). An aliquot of fraction 2 F (68.7 mg) was subjected to HPLC (YMC-Pack SIL-06, AcOEt-MeOH = 7:1, flow rate 1.0 mL/min) to afford fractions 8 A to 8F. Fraction 8B (12.4 mg) was subjected to Sephadex LH-20 column chromatography (φ 15 × 400 mm; MeOH) to give compound **7** (0.8 mg). An aliquot of fraction 2A (192.6 mg) was subjected to Sephadex LH-20 column chromatography (φ 30 × 420 mm; MeOH) to afford fractions 15A to 15E. Fraction 15B (15.7 mg) was subjected to ODS HPLC (COSMOSIL 5C_18_-AR-II; 40% MeOH + 0.1% HCOOH, flow rate 3.0 mL/min) to give compound **8** (7.0 mg), compound **9** (2.0 mg) and compound **10** (0.8 mg).

*Terminalia chebula* (bark; 395 g) MeOH extract was partitioned with hexane, EtOAc and BuOH to obtain hexane (1.5 g), EtOAc (3.6 g), BuOH (42.6 g) and aqueous (20.5 g) soluble fractions. The EtOAc fraction containing the target peak was subjected to silica gel column chromatography (φ 50 × 250 mm; CHCl_3_-MeOH = 1:0–0:1) to give fractions 1A-H. Fraction 1D contained the target peak and was separated by ODS column chromatography (φ 25 × 200 mm; MeOH-H_2_O = 7:3–0:1) to give fractions 2A–F. Fraction 2A was subjected to ODS column chromatography (φ 20 × 200 mm; MeOH-H_2_O = 7:3-0:1) to give fractions 6A–G. Fraction 6E was separated by reverse-phase HPLC (CAPCELL PAK C_18_ MGII; 50% MeOH) to give compound **11** (0.4 mg). Compound **11** was identified as 4-*O*-(4″-*O*-galloyl-α-L-rhamnopyranosyl)ellagic acid by comparison of its NMR data with reported spectral data.

### Cell culture

C17.2 cells were cultured in proliferation medium [DMEM (DS Pharma Biomedical Co., Ltd. Osaka, Japan) supplemented with 10% fetal bovine serum (Biowest, Nuaillé, France), 5% horse serum (Gibco, Life Technologies, Tokyo, Japan)]. Neural differentiation of C17.2 cells was initiated by transferring the cells to differentiation medium [Neurobasal Medium (Gibco) supplemented with 2% B-27 without vitamin A (Gibco), 1% antibiotic-antimycotic (Gibco)]. All cultures were maintained in a humidified incubator at 37 °C in 5% CO_2_/95% air.

### Neural stem cell differentiation assay

C17.2 cells were dissociated using the proliferation medium 2 × 10^5^ cells/mL on cover glass (MATSUNAMI GLASS) coated with poly-L-lysine and fibronectin/laminin. After incubation for 24 h, cells were washed with D-MEM and treated with individual compounds in differentiation medium for 6 days.

### Immunofluorescence staining

C17.2 cells were fixed with 4% paraformaldehyde in PBS for 20 min at room temperature (r.t.) and washed with 1% BSA in PBS three times. After blocking with 10% BSA in 0.3% Triton X-100 in PBS for 45 min at r.t., fixed cells were incubated with primary antibodies (anti-βIII-Tubulin; Neuronal Class III, Mouse-Mono, 1:400, R&D Systems^TM^; anti-GFAP, 1:400, VERITAS) for 12 h at 4 °C. After washing three times with 1% BSA in PBS (500 μL/well), cells were incubated with secondary antibodies (Alexa Fluor^®^ 488 goat anti-mouse IgG (H + L), 1:400, Life Technologies; Alexa Fluor^®^ 555 goat ant-rabbit IgG (H + L), 1:200, Life Technologies) for 1 hr at r.t. in the dark. After washing three times with 1% BSA in PBS (500 μL/well), cells were treated with 200 μg/mL RNase (Nacalai Tesque) in PBS containing 1% BSA and 0.1% Triton X-100 for 1 hr at 37 °C in the dark. After washing three times with 500 μL/well PBS, cells were incubated in 30 μM TO-PRO^®^-3, 1% BSA, and 0.1% Triton X-100 in PBS for 10 min at r.t. in the dark. The cover glass on which the cells were attached was transferred onto slide glass and mounted with ProLong^®^ Gold Antifade Reagent with DAPI (Invitrogen). Slide glasses were viewed and photographed with an LSM 780 (Carl Zeiss). Four pictures were taken per well, and the assays were carried out with three individual wells per condition.

### *In silico* analysis

The initial 3D structures of 4-*O*-(4″-*O*-galloyl-α-L-rhamnopyranosyl)ellagic acid were constructed using Build in MOE (version 2016; Chemical Computing Group, Montreal, Canada) with standard geometric parameters. The ligand was then minimized using Energy Minimize in MOE with the Amber force field until the root-mean-square (rms) energy gradient was less than 0.001 kcal mol^−1^Å^−1^. The protein model was constructed based on the structure of HLH domain taken from the Protein Data Bank (PDB) (PDB code: 2MH3)^38^. Water molecules in the structure were removed. All hydrogen atoms were added and Amber all-atom charges were assigned for the whole protein. The molecular docking simulations were performed using the Induced Fit methods in MOE-Dock.

## Supplementary information

Supplementary Info.
