# Supplementary material for: Target protein-oriented isolation of Hes1 dimer inhibitors using protein based methods
Source: Sci Rep. 2020 Jan 28;10:1381. doi: 10.1038/s41598-020-58451-3 (PMC6987128; doi:10.1038/s41598-020-58451-3)
Supplement: Supplementary file 1 — Supplementary Info. [file 41598_2020_58451_MOESM1_ESM.docx]

**Supporting Information**

**Target protein-oriented isolation of Hes1 dimer inhibitors using protein based methods**

*Midori A. Arai,^*^ Kaori Morita, Haruka Kawano, Yuna Makita, Manami Hashimoto, Akiko Suganami, Yutaka Tamura, Samir K. Sadhu, Firoj Ahmed, and Masami Ishibashi^*^*

**Expression and purification of recombinant GST fused and cleaved proteins**

*E. coli* strain JM109 (Nippon Gene Co., Ltd., Tokyo, Japan) serves as hosts for pGEX-ratHes1_3-281_, pGEX-ratHes1_1-95_, pGEX-hFANCF_1-374_, which plasmids described previously.^1^ JM109 was cultured overnight to plateau phase and inoculated to fresh LB medium (Invitrogen) containing 100 mg/L ampicillin. Cells were grown at 37 ˚C to 0.6 (OD_600_) and induced of protein synthesis by addition of 1 mM IPTG (isopropyl-1-thio-β-D-galactopyranoside) followed by incubation for an additional 6 h at 18 ˚C. The cells were harvested by centrifugation and lysed by sonication. The lysate was centrifuged at 6000 rpm for 5 min at 4 ˚C. After incubating with 1% Triton X-100 for 30 min, the lysate was centrifuged at 7000 rpm for 7 min at 4 ˚C. The resulting supernatant was added to pre-washed glutathione sepharose 4B beads (GE Healthcare, Uppsala, Sweden) and gently mixed at 4 ˚C for 1 h. The beads obtained after centrifugation (2000 rpm, 5 min, at 4 ˚C) were washed 5 times (2000 rpm, 1 min, at 4 ˚C) with WE buffer (20 mM Tris-HCl, pH 7.5, 2 mM MgCl_2_, 1 mM DTT). The GST was cleaved from recombinant protein (GST-Hes1_3-281_) by Turbo3C Protease (Accelagen Inc., San Diego, USA) for 4 h at 4 ˚C in cleavage buffer (50 mM Tris-HCl, pH 7.5, 150 mM NaCl, 1 mM EDTA, 1 mM DTT), then dialyzed against PBS buffer using Slide-A-Lyzer^®^ Dialysis Cassette (Extra Strength, 10,000 MWCO; Thermo, Rockford, USA). For GST fused proteins, the recombinant proteins were eluted with 50 mM glutathione buffer (50 mM Glutathione (Reduced Form), 50 mM Tris-HCl, pH 8.8) and then dialyzed against PBS buffer. The protein concentration was determined by Micro BCA Protein Assay Kit (Thermo, Rockford, USA). The recombinant proteins were estimated to be greater than 90% pure by SDS-PAGE.

Ref1: Arai, M. A.; Uemura, K.; Hamahiga, N.; Ishikawa, N.; Koyano, T.; Kowithayakorn, T.; Kaddar, T.; Carreau, M.; Ishibashi, M. "Naturally occurring FANCF-Hes1 complex inhibitors from *Wrightia religiosa*" *Med. Chem. Commun.* **2015**, *6*, 455-460.

**Preparation of Hes1 bound microplate.**

Typical procedure; Nunc Immobilizer^TM^ Amino 96 well Plate, white (Nalge Nunc Int., NY, USA) was used for immobilizing of Hes1. The micro plate wells were incubated with 100 μl of Hes1 (10 μg/mL in PBS) 2 h at 4 ˚C. After removing protein solution, the wells were incubated for 2 h at 4 ˚C with 100 μL of 10 mM ethanolamine (in 100 mM Na2CO3 buffer, pH 9.6) to block remaining activated units on the well. Then, wells were washed twice with 200 μL of PBST (PBS containing 0.05% Tween 20).

**Preparation of Cy3-labeled Hes1 protein.**

The Cy3 dye reagent was prepared as 1 vial of Cy3 monofunctional dye reagent (succinimidylester type; GE Healthcare, Buckinghamshire, UK) in DMSO (50 μL) and stored at -20 ˚C. Cy3-labeled Hes1 was prepared by mixing of protein in PBS with Cy3 dye reagent in DMSO (typically 0.6 μL of DMSO solution to 1 mL of 10 mg/L protein solution in PBS) for 30 min at 4 ˚C in the dark. After incubation, the mixture was dialyzed with PBS buffer at 4 ˚C for 3 h to remove excess unreacted dye reagent, then with NET buffer (20 mM Tris-HCl, pH 7.5, 200 mM NaCl, 1 mM EDTA) at 4 ˚C for overnight to replace buffer. The ratio of dye/protein was calculated from the absorption for Cy3 (ε 150000 M^-1^cm^-1^ at 552 nm).

**Plate assay for Hes1 dimer inhibitors.**

The Hes1 bound microplate wells were incubated with 50 μL of Cy3-labeled-Hes1 in NET-N buffer (NET buffer containing 0.05% Nonidet^®^ P-40, ca. 7 mg/L, dye/protein = 0.4) for 24 h at 4 ˚C. After removal of protein solution, wells were washed twice with 200 μL of PBST. Then, each compound solution (NET-N buffer, 50 μL) was added and incubated for 1 h at RT in the dark. Wells were washed twice with 200 μL of PBST, then dried under reduced pressure for 1 h in the dark. The fluorescence intensity was detected by a microplate reader (Fluoroskan Ascent, Thermo Labsystems, Vantaa, Finland). The Cy3 dye was excited at 544 nm and emitted at 590 nm. Usually, the assays were carried out in three individual wells, and the mean value and SD were calculated. There would be the equilibrium of the dimmer formation between Cy3-Hes1/Cy3-Hes1 (in solution) and Cy3-Hes1/immobilized-Hes1. As the results, Cy3-Hes1/immobilized-Hes1 can be detected.

**Figure S1** Isolation scheme of *Psidium guajava*

**Figure S2** HPLC Chart of isolated compounds (**1**-**10**)

**Figure S3.** The HPLC results of screening. GST-Hes1 beads and GST beads.

**Figure S4** Isolation scheme of *Terminalia chebula*

**Figure S5** HPLC Chart of isolated compound **11**

**Table 1** Spectroscopic data of isolated compounds
